# Initial treatment for surgery-naïve desmoid tumors by high intensity focused ultrasound

**DOI:** 10.3389/fonc.2024.1388302

**Published:** 2024-07-22

**Authors:** Jiayi Shen, Jing Zhao, Xian Zhong, Shuyi Xie, Lanqi Wu, Chenlu Hu, Xiaoye Hu, Hong Shen

**Affiliations:** ^1^ Department of Medical Oncology, The Second Affiliated Hospital, Zhejiang University School of Medicine, Hangzhou, Zhejiang, China; ^2^ Zhejiang University-University of Edinburgh (ZJU-UOE) Institute, Zhejiang University School of Medicine, Zhejiang University, Haining, Zhejiang, China; ^3^ College of Basic Medical Sciences, Tianjin Medical University, Tianjin, China

**Keywords:** high-intensity focused ultrasound ablation (HIFU), low power cumulative, desmoid tumor (aggressive fibromatosis), initial treatment, abdominal wall desmoid tumor, extra abdominal desmoid tumor

## Abstract

**Introduction:**

Desmoid tumor (DT) is a rare proliferative disease occurring in connective tissues, characterized by high infiltration and recurrence rates. While surgery remains the primary treatment, its recurrence risk is high, and some extra-abdominal desmoid tumors are inoperable due to their locations. Despite attempts with radiotherapy and systemic therapy, the efficacy remains limited.

**Methods:**

We used low-power cumulative high-intensity focused ultrasound (HIFU) therapy as an initial treatment for desmoid tumor patients either ineligible or unwilling for surgery. Low-power cumulative HIFU employs slower heat accumulation and diffusion, minimizing damage to surrounding tissues while enhancing efficacy.

**Results:**

Fifty-seven non-FAP desmoid tumor patients, previously untreated surgically, underwent low-power cumulative HIFU therapy. Among them, 35 had abdominal wall DT, 20 had extra-abdominal DT, and 2 had intra- abdominal DT, with an 85% median ablation ratio. Abdominal wall DT patients showed significantly better response rates (91.4% vs. 86%) and disease control rates (100% vs. 32%) than that of non-abdominal wall DT patients. Median event- free survival time was not reached after a median follow-up duration of 34 months.

**Discussion:**

With its high response rate, durable efficacy, and mild adverse effects, our findings suggest that low-power cumulative HIFU presents a promising novel treatment for desmoid tumors, particularly abdominal wall DT patients.

## 1 Introduction

Desmoid tumor (DT), also known as aggressive fibromatosis, is a rare monoclonal, locally invasive, non-metastasizing, usually unifocal fibroblastic proliferative disease with complex etiology (1). It occurs in connective tissues such as the abdominal wall, mesentery, and limbs (2). The incidence is about 3 cases per one million individuals per year, with a peak age of 30-40 years and a predominance in females (3). Though desmoid tumors have a low incidence and are generally considered benign, they are still worth attention due to their high potential for infiltration and recurrence (4). Being able to infiltrate into adjacent tissues, DT could lead to chronic pain, functional impairment, and sometimes life-threatening conditions (1). Based on etiology, DT is categorized into two main types: sporadic DT (85-90%) and familial adenomatous polyposis (FAP)-associated DT (10-15%) caused by germline mutations in the APC (adenomatous polyposis coli) gene (3). Risk factors of DT include trauma, surgery, and pregnancy (2).

Based on the location of the tumor, DT can be classified into three categories, extra-abdominal DT, abdominal wall DT, and intra-abdominal DT. Extra-abdominal DT occurs in the upper body, most frequently the upper arm with an approximate occurrence of 30% (5). When the tumor appears in the head or neck, it can infiltrate around the axillary vessels, brachial plexus, and airways, restricting surgical resectability (6). Abdominal wall DT generally occurs in women of childbearing age and can occur after pregnancy (7). It is usually solitary and slow-growing, often presented and detected early (5). Intra-abdominal DT tends to occur in the pelvis, mesentery, and retroperitoneum, most commonly in the mesentery of the small intestine (5). It is complicated by small intestinal fistula, abscess formation, gastrointestinal bleeding, and intestinal obstruction.

Although local treatments like surgery and radiotherapy are commonly used in treating DT, there is no unified standard for treating DT. Surgery (local control rate of 61%) is commonly used for treating operable DT if there is a clear margin to follow. However, a high local recurrence (25-60%) has been reported even after complete excision (8). Radiotherapy (local control rate of 78-81%) is applied as supplementary therapy or for inoperable DTs but it is time-consuming and may cause a range of post-treatment complications such as nerve and skin damage (4, 9). New systemic treatments for DT have shown high response rates. These include the first approved drug for DT, nirogacestat (10), which is a reversible γ-secretase inhibitor targeting Notch signaling, and sorafenib (11), a tyrosine kinase inhibitor. However, long-term adverse effects of these drugs, particularly on female reproductive function, are still not well understood (12).

High intensity focused ultrasound (HIFU) is a novel, non-ionizing, and non-invasive ablation technique that minimizes the risks and complications of invasive procedures. Focusing ultrasound beams, HIFU can generate a well-defined area of coagulative necrosis by elevating temperature rapidly to more than 60°C in the targeted tissue, while minimizing damage to the adjacent normal structures (13). It has been applied in treating prostate, breast, and brain tumors and is considered a promising therapeutic approach to treat recurrent DT because it is safe and repeatable (14).

The traditional ultrasound-guided HIFU technique uses high input power and short pulse duration to accumulate heat quickly in the targeted location. Damage to the surrounding normal tissue is indeed a concern because of the transient high power. A novel HIFU technique called low-power cumulative HIFU which uses low input power and long emissions was introduced by Zhao et al. in 2017 (15). The speed of heat accumulation and diffusion is much slower as compared to the traditional ultrasound-guided HIFU approach. Therefore, higher efficacy of heat accumulation would be achieved while less normal tissues would be damaged (16). Since then, low-power cumulative HIFU has been applied to treat many diseases including pancreatic cancer (15) and DT (8). It is found that low-power cumulative HIFU could effectively increase the survival rate and reduce adverse effects (15).

We recently reported a cohort of 91 patients with recurrent desmoid tumors who received low-power cumulative HIFU treatment after surgical failure (16). However, no previous study has shown the effect of HIFU as an initial treatment on DT patients. In this retrospective study, we collected data from 57 patients who received low-power cumulative HIFU treatment in the Department of Oncology, the Second Affiliated Hospital, Zhejiang University, School of Medicine between July 2015 and October 2022. 35 patients with abdominal wall DT chose HIFU treatment when they were offered to choose between surgery and HIFU. Surgery is a traditional treatment plan for abdominal wall DT patients and here we propose that low-power cumulative HIFU treatment could be an alternative option with less invasiveness and good survival outcomes for those patients. 22 patients with extra-abdominal and intra-abdominal DT were incapable of surgery because of the locations and the sizes of tumors. All patients had non-FAP-associated DT and did not receive surgical treatments for DT before low-power cumulative HIFU treatment. So far as we knew, this is the first and largest cohort of initial therapy of low-power cumulative HIFU in treating DT. Here we present our experience and results of treating those patients.

## 2 Materials and methods

### 2.1 Patients

Patients with DT who received HIFU treatments between July 2015 to October 2022 in the Department of Medical Oncology, the Second Affiliated Hospital, Zhejiang University, School of Medicine were retrospectively collected. The inclusion criteria were: (1) histological diagnosis criteria of DT including no morphology amorphism, aggressive growth of striated muscle, no collagen, positive nuclear staining for β-catenin (17); (2) no previous surgeries for DT; (3) tumor adequately visible on ultrasound; (4) Karnofsky performance status (KPS) > 50. Patients fulfilling these criteria were excluded from this study: (1) non-eligible for general anesthesia; (2) tumor not adequately visible on ultrasound; (3) extensive scarring along the acoustic path. Patients are actively observed before treatment and HIFU was performed when tumor enlargement was observed. The median time from histological diagnosis to HIFU treatment was 2 months. All 57 are not FAP-associated DT patients and accepted low-power cumulative HIFU in our center. Informed consent was obtained from each patient. The study was approved by the ethics committee of the Second Affiliated Hospital, Zhejiang University, School of Medicine (Case number 2020-831).

### 2.2 Device and treatment plan

HIFU treatment was performed with anesthesia using the FEP-BY02 system (Beijing Yuande Biomedical Engineering, China) and guided in real-time by a diagnostic ultrasound device (Logic 5, GE Healthcare, USA). For real-time guidance during HIFU, the imaging probe (3.5 MHz) was integrated into the center of the HIFU transducer. Parameters for HIFU were as follows: ultrasonic power: 100-300 W (input power varied depending on the tumor depth); emission time of T1: 990 ms; interval time of T2: 10 ms; the total transmissions for each therapeutic point: 40 times (2 mm between adjacent points). Each treatment unit generally consists of 3 to 5 therapeutic points, with a spacing of 2 mm between each unit, and a spacing of 5 mm between adjacent slices. A section may include several treatment units, and with time, heat accumulation occurs, causing expansion of the ablation boundary. Contrast-enhanced ultrasound was utilized repeatedly during treatment to evaluate ablation progress. The determination of whether the ablation boundaries of ultrasonography were safe and sufficient and whether the goal of treatment was achieved was based on the judgment of the treating physician (see **Supplementary Figure 1**). The total emission lasted < 90 min, and the entire therapeutic schedule lasted 1-2 hours, depending on the tumor size and location.

During our low-power cumulative HIFU therapy, the real-time changes of the targeted lesion were monitored using ultrasound (B-mode ultrasound). It was common to repeat HIFU ablation multiple times for a single tumor lesion because we adopted a conservative strategy and did not ablate the entire tumor in a single session. Additionally, ultrasound (B-mode ultrasound used here) does not provide clear visualization of desmoid tumor’s boundaries. After each session, treatment boundaries were marked by MRI and response evaluation was done.

The treatment process was repeated once every 3-4 weeks until the maximum ablation ratio for the patient was achieved based on MRI examination result. The intervals between sessions were necessary because heat diffusion occurs after each treatment session, requiring time for stabilization. Patients were carefully observed for recurrence and possible complications such as skin burns, nerve injury, and perforation.

### 2.3 Observation and measurement

Ultrasound and contrast-enhanced MRI were used to examine the treated area one month after completing the HIFU procedure. Tumor lesions where no enhancement was observed after contrast-enhanced MRI were identified as necrotic tissues, while those with enhancement were considered viable tumors. The ablation ratio was calculated as the maximal necrotic tumor diameter divided by the maximal tumor diameter. The maximum diameters of tumor(s) before and 1-month after HIFU treatment were recorded. For patients with desmoid tumors in multiple sites, the maximum diameter was calculated as the sum of all tumors. The best overall therapeutic response was evaluated by contrast-enhanced MRI, using Response Evaluation Criteria in Solid Tumors (RECIST) 1.1. Follow-ups were carried out every 4-6 months after the first HIFU treatment until December 2022.

## 3 Results

Among the 57 patients, 35 with abdominal wall DTs chose HIFU over surgery. The remaining 20 patients had extra-abdominal or intra-abdominal DTs on sites where surgeries were incapable, including the chest wall, neck, and shoulder.

Patients who received the low power cumulative HIFU treatment are mostly females (55 females, 2 males) and their ages range from 19 to 69 with a median of 32. There are 20 patients with extra-abdominal DT, 35 cases with abdominal wall DT, and 2 intra-abdominal. Among the 35 patients with abdominal wall DT, 33 were females and 27 had a pregnancy history, which is consistent with the prevalence characteristics of this type of tumor (7). 3 extra-abdominal DT patients had tumors at multiple locations and the maximum tumor diameter of all patients before HIFU treatment ranges from 1.3 cm to 20.0 cm with a median of 7.0 cm.

**Figure 1:**
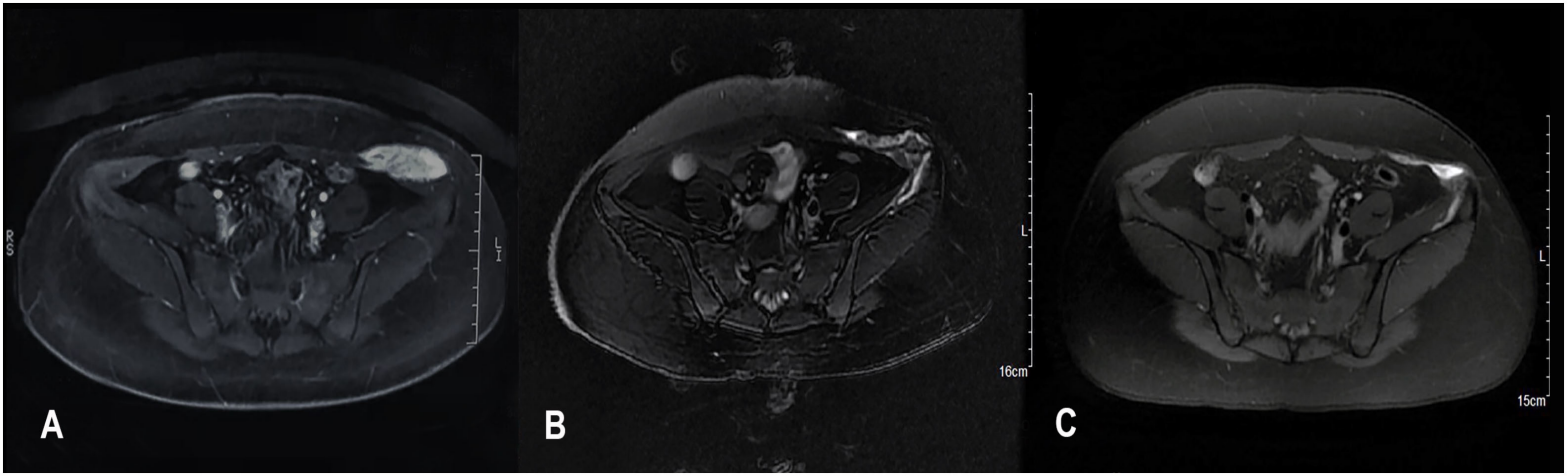
MRI images from before **(A)** and 3 months after **(B)** and 6 months after **(C)** HIFU therapy. **(A)** A 4.5 cm tumor located at the left lower quadrant; **(B)** The tumor regressed significantly from 4.5cm to 1.5 cm in diameter with obvious necrosis inside; **(C)** The tumor was significantly necrotic and absorbed. The MRI sequence is T2 FLAIR.

As an example, a 29-year woman was referred to our hospital for a painful left abdominal wall mass and diagnosed as DT. A 4.5cm tumor located at the left lower quadrant was detected by MRI (**Figure 1A**) and diagnosed as DT after aspiration biopsy. She underwent 3 HIFU treatments. On the contrast-enhanced MRI three months after the last treatment, the tumor regressed significantly from 4.5cm to 1.5cm in diameter with obvious necrosis inside (**Figure 1B**). Six months later, the MRI showed patchy mixed signals with an unclear margin in oblique internal abdominal muscle, which demonstrated that the tumor was significantly necrotic and absorbed (**Figure 1C**). By now, no tumor recurrence was observed.

The median ablation ratio was 85%. After the median follow-up duration of 34 months (range 4-75 months), the largest diameter of abdominal wall DT patients decreased by 74% on average while that of non-abdominal wall DT patients decreased by 11% (**Table 1**).

**Table 1:** Overall curative effects.

ID	Gender	Age	Tumor Location	Maximum tumor diameter before treatment (cm)	Number of treatments	Ablation ratio (%)	Reduction ratio of the largest diameter (%)	Response evaluation	Follow-up duration (months)
1	F	23	Abdominal wall	7.0	3	100%	0%	CR	75
2	M	20	Abdominal wall	9.0	6	78%	30%	PR	58
3	F	28	Abdominal wall	7.0	3	100%	0%	CR	57
4	F	31	Abdominal wall	8.0	4	96%	25%	PR	56
5	F	30	Abdominal wall	5.0	3	100%	0%	CR	42
6	F	19	Extra-abdominal	11.0	8	54%	140%	PD	17
7	F	27	Abdominal wall	7.0	6	86%	17%	PR	47
8	F	22	Abdominal wall	4.0	2	95%	0%	CR	45
9	F	36	Abdominal wall	5.5	3	100%	33%	PR	24
10	F	28	Abdominal wall	7.0	4	88%	40%	PR	47
11	F	31	Extra-abdominal	6.0	5	74%	NA	PD	NA
12	F	33	Abdominal wall	8.0	4	93%	24%	PR	35
13	F	33	Abdominal wall	8.5	3	97%	0%	CR	30
14	F	34	Extra-abdominal	16.0	3	85%	68%	PR	36
15	F	38	Extra-abdominal	14.0	4	53%	104%	SD	36
16	F	32	Abdominal wall	7.4	5	80%	52%	PR	37
17	F	32	Abdominal wall	7.0	5	90%	14%	PR	34
18	F	32	Extra-abdominal	11.0	9	70%	29%	PR	34
19	F	35	Extra-abdominal	5.6	5	20%	100%	SD	37
20	F	30	Abdominal wall	14.0	10	80%	43%	PR	21
21	F	32	Abdominal wall	6.6	4	90%	0%	CR	24
22	F	30	Abdominal wall	8.1	3	90%	26%	PR	24
23	F	28	Abdominal wall	5.7	4	90%	0%	CR	29
24	F	28	Extra-abdominal	10.2	4	70%	65%	PR	19
25	F	29	Abdominal wall	4.0	2	90%	48%	PR	4
26	F	33	Abdominal wall	5.0	2	50%	74%	SD	23
27	F	32	Abdominal wall	7.0	2	100%	16%	PR	19
28	F	31	Extra-abdominal	8.0	6	60%	75%	SD	23
29	F	52	Extra-abdominal	6.1	6	30%	105%	SD	24
30	F	35	Abdominal wall	8.7	3	90%	43%	PR	22
31	F	34	Abdominal wall	3.0	2	90%	33%	PR	15
32	F	34	Abdominal wall	2.6	3	80%	69%	PR	15
33	F	21	Abdominal wall	4.7	2	90%	38%	PR	14
34	F	31	Abdominal wall	5.3	4	70%	57%	PR	11
35	F	27	Extra-abdominal	6.4	1	80%	33%	PR	12
36	F	39	Extra-abdominal	6.6	3	90%	90%	SD	6
37	F	32	Extra-abdominal	13.5	6	10%	89%	SD	7
38	F	28	Abdominal wall	2.5	2	70%	84%	SD	7
39	F	28	Abdominal wall	5.0	0	100%	0%	CR	69
40	F	35	Abdominal wall	10.0	6	73%	60%	PR	43
41	F	29	Abdominal wall	5.0	0	90%	0%	CR	59
42	F	38	Abdominal wall	15.0	4	91%	27%	PR	59
43	F	30	Abdominal wall	4.0	0	100%	0%	CR	55
44	F	29	Intra-abdominal	12.0	11	72%	92%	SD	54
45	F	41	Extra-abdominal	4.0	1	89%	25%	PR	51
46	F	44	Abdominal wall	4.0	0	100%	0%	CR	51
47	F	69	Extra-abdominal	10.0	13	85%	130%	PD	49
48	F	25	Extra-abdominal	10.0	9	74%	90%	SD	48
49	F	29	Abdominal wall	5.0	3	66%	50%	PR	47
50	F	39	Intra-abdominal	10.0	9	39%	92%	SD	42
51	F	34	Extra-abdominal	8.0	2	22%	88%	SD	55
52	F	31	Abdominal wall	7.2	2	90%	0%	CR	36
53	F	38	Extra-abdominal	8.0	4	50%	63%	PR	28
54	M	67	Abdominal wall	7.1	3	70%	92%	SD	7
55	F	34	Extra-abdominal	9.5	3	60%	89%	SD	24
56	F	24	Extra-abdominal	20.0	4	70%	110%	SD	18
57	F	66	Extra-abdominal	1.3	1	90%	62%	PR	18

The ablation ratio is a short-term outcome calculated as the maximal ablated diameter over the overall maximal tumor diameter, which was obtained 1 month after the therapeutic process through MRI. The reduction ratio of the largest diameter is a final result defined as the value of the maximum tumor diameter at the latest follow-up session divided by the largest diameter before HIFU treatment. Maximum tumor diameter numbers were obtained by MRI after therapeutic sessions. (CR, complete response; PR, partial response; SD, stable disease; PD, progressive disease); NA, not available.

The therapeutic responses have significant differences between abdominal wall DT patients and non-abdominal wall DT patients. For abdominal wall DT patients (**Table 1**), the response rate was 91.4% (32/35), among which 12 patients achieved complete responses 1.4 years on average after low-power cumulative HIFU treatment. Surprisingly, only 8.6% (3/35) of abdominal wall DT patients remained in stable disease states, among which one developed SD after he paused HIFU treatment for 7 months and was later treated with surgery. The disease control rate of abdominal wall DT patients was 100%. In comparison, extra-abdominal and intra-abdominal DT patients had a worse response with a disease control rate of 86% (**Table 1**). None of these patients achieved complete responses 30 months on average after HIFU treatment, and 32% (7/22) achieved partial responses. 54% (12/22) of these patients had stable diseases while 14% (3/22) developed progressive diseases.

**Figure 2:**
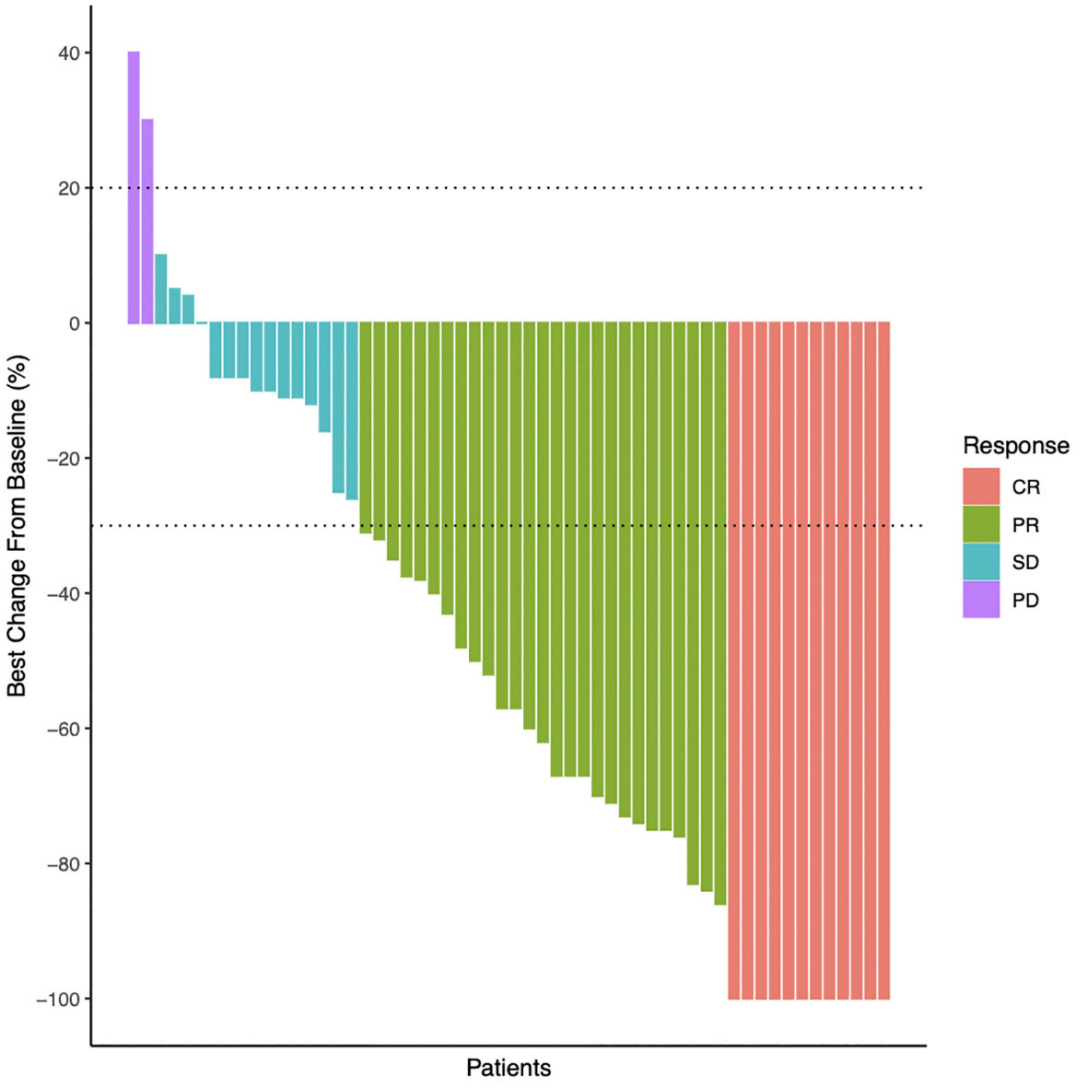
Best percentage change from baseline in tumors maximum diameter. Waterfall plots of the treatment response in terms of the greatest change in tumor maximum diameter compared with the pretreatment diameter.

Although there is variation between individuals and tumor types, it was found that the greatest change in tumor diameter is negatively correlated with the treatment response (**Figure 2**).

**Figure 3:**
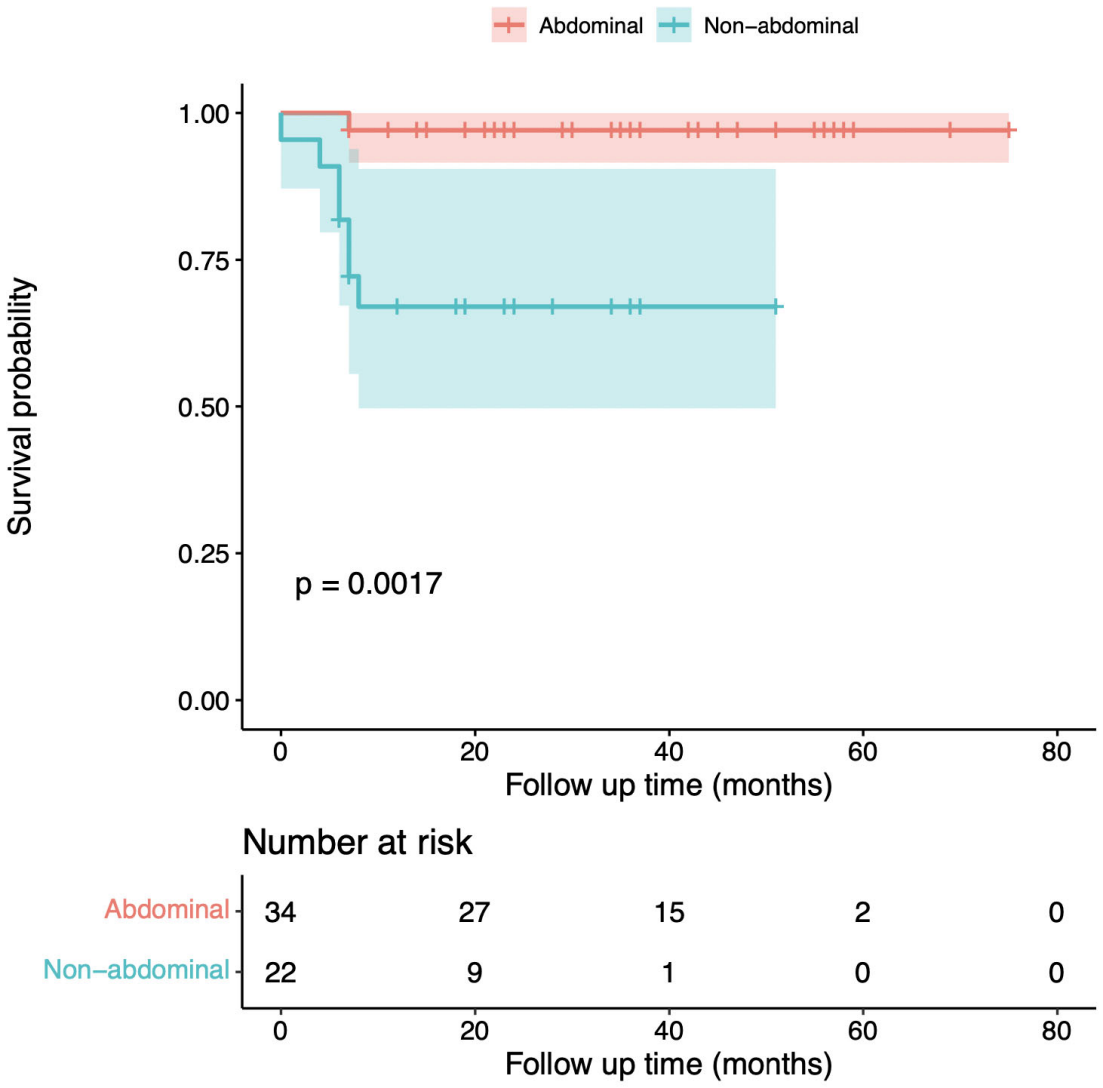
Event-free survival rate of abdominal wall desmoid tumor patients and non-abdominal wall desmoid tumor patients. The Kaplan-Meier plots of event-free survival outcomes of abdominal wall DT patients (red) and extra- and intra-abdominal DT patients (green). The shaded area represents 95% confidence intervals. One patient (patient ID 11) was lost to follow-up after the first session of treatment as indicated in **Table 1**.

Follow-ups were conducted until December 2022. Here, we measure event-free survival until the time when the tumor enlarges and requires further supplemental therapy. During a median follow-up duration of 34 months (range from 4 to 75 months), the 5-year event-free survival rate was 97% for abdominal wall desmoid tumor patients and 68.2% for extra- and intra-abdominal DT patients (**Figure 3**). All patients who achieved complete tumor necrosis remained event-free for 30 months on average (**Table 1**). One of the patients, a 30-year-old woman, successfully gave birth to a baby after HIFU treatment.

The side effects were mild in this study. Four patients had grade 1 skin burns and recovered soon. One patient suffered from a grade 1 nerve injury but recovered completely 3 months later. No other adverse effects including low-grade fever, thrombocytopenia, and perforation were found in this study.

## 4 Discussion

Surgery is the main therapeutic approach for DT; however, it is not an ideal way for those patients with tumors that are overly large or affect adjacent critical structures. Furthermore, the post-surgery recurrence rates are high. Penel’s prospective study which enrolled 771 DT patients demonstrated the 2-year EFS as 53% in the surgery group and no significant differences were observed between the surgery group and the observing group (18). One of the reasons is that surgery as an iatrogenic trauma may be one of the causes of fibromatosis. Given the rate of relapse after surgery, there has been a shift to a more conservative approach, namely the “wait-and-see policy” (19, 20). The National Comprehensive Cancer Network (NCCN) guideline of soft tissue sarcoma (Version 2.2021) has recommended observation as an option for selected patients whose tumors are not symptomatic, impairing, or threatening in function (4). However, more than 40% of patients managed by the “wait-and-see” approach experience disease progression (18, 21). Radiotherapy is another treatment option. In a phase II trial of moderate dose radiotherapy (56 Gy in 28 fractions) for inoperable DT, the overall response rate was 50% and the 3-year local control rate was 81.5% in 44 patients (9). Toxicity of skin, mucosal membranes, and pain are the common acute side effects that might occur. However, late toxicity such as post‐irradiation fibrosis, joint contracture, and neuropathy might seriously affect the quality of life (4). The risk of a second primary tumor in the relatively young population deserves more attention (22). A non-invasive, high-efficiency, and well-tolerated local treatment approach is urgently needed.

HIFU is a non-invasive method that uses ultrasound beams to focus on the target tissue specifically, destroying the tumor tissues by two known mechanisms, heat and cavitation effects (23). It has been approved by the China Food and Drug Administration (CFDA). Previous research has shown that HIFU can be used in treating DT patients with recurrence after surgery (8, 24, 25) with a favorable prognosis. In these studies, HIFU treatment more often served as a salvage approach for recurrent desmoid tumors. No previous studies reported HIFU as an initial treatment in DT patients. Traditional ultrasound-guided HIFU therapy usually adopts a high input power (400-1000w) and short pulse duration (150-200ms) protocol, which causes a transient elevating temperature of the target lesion. This model would release high thermal energy over a short time which would easily damage the adjacent normal tissues. In this study, we introduced a HIFU treatment called low-power cumulative HIFU, which has been used in our medical center for several years. This model uses lower input power (100-300w) and prolonged pulse duration (990ms), which make the temperature in the target area increase slowly, maximizing the effect of heat accumulation and reducing the damage to adjacent normal tissue.

In this study, all the DT patients were not capable of surgery or refused surgery and accepted low-power cumulative HIFU therapy as first-line therapy. After the low-power cumulative HIFU treatment, the ablation ratio ranged from 10% to 100%. 49 out of 58 patients’ tumors regressed and no further tumor growth has been observed during the follow-up period. The efficacy was durable and persistent. It is interesting to note that some patients’ tumors continue to shrink one or two years after HIFU treatment.

The treatments were tolerated by most patients. Four patients suffered from skin burns and one suffered from nerve injury but all recovered quickly after proper treatment. Those patients had large tumors with maximum diameters of 8-20 cm, 2 with abdominal wall DT, and 3 with extra-abdominal DT. Those tumors showed poor responses during treatment, only with 63% of tumor size ablated on average and 3 patients were evaluated as SD. These cases suggest large volumes and intricate anatomical locations lead to poor response tumors. Simply increasing output power does not improve the curative effect but causes side effects. Optimizing HIFU treatment parameters and times is the key issue. HIFU can be easily accepted by patients because it is non-invasive with low adverse event rates. Patients could recover from the operation quickly. If the tumor recurs or progresses, HIFU could be performed repeatedly because there is no accumulated toxicity. Within the study, one patient received systematic treatment during HIFU therapy aiming to decelerate tumor growth.

So far as we know, this is the first cohort of DT patients who received low-power cumulative HIFU treatment as an initial treatment. Our data especially showed that for abdominal wall desmoid tumor patients, low-power cumulative HIFU could be performed as a new curative treatment with non-invasiveness, high response rates, long-lasting efficacy, and low adverse effects. The 5-year event-free survival rate for abdominal wall DT patients (97%) was higher than a cohort of patients who underwent surgery (90%) (26). Here, we suggest a possible therapeutic strategy that abdominal wall desmoid tumor patients could be treated with low-power cumulative HIFU instead of surgery after biopsy diagnosis.

There are some limitations to this study. First, this was a single-center retrospective study. Second, the follow-up time may not be long enough to observe tumor recurrence. We would continue to follow up, and we look forward to a larger and multi-center clinical trial to validate the survival benefit of HIFU treatment.

## 5 Conclusions

In summary, we report the results of a therapeutic strategy using low-power cumulative HIFU to initially treat desmoid tumor patients which is especially suitable for abdominal wall DT patients, showing high disease control rates and good survival outcomes. This strategy is also recommended for treating non-abdominal wall DT patients with inoperable tumors.

## Data availability statement

The original contributions presented in the study are included in the article/**Supplementary Material**. Further inquiries can be directed to the corresponding authors.

## Ethics statement

The studies involving humans were approved by The Second Affiliated Hospital, Zhejiang University, School of Medicine. The studies were conducted in accordance with the local legislation and institutional requirements. The participants provided their written informed consent to participate in this study.

## Author contributions

JS: Data curation, Formal analysis, Methodology, Validation, Visualization, Writing – original draft, Writing – review & editing. JZ: Data curation, Writing – review & editing. XZ: Data curation, Writing – review & editing. SX: Formal analysis, Writing – original draft. LW: Formal analysis, Writing – original draft. CH: Data curation, Validation, Writing – review & editing. XH: Conceptualization, Investigation, Methodology, Project administration, Supervision, Visualization, Writing – review & editing. HS: Conceptualization, Investigation, Methodology, Project administration, Supervision, Visualization, Writing – review & editing.

## Supplementary material

Supplementary Figure 1 Contrast-enhanced intraoperative ultrasound imaging used for intraoperative ablation monitoring. Ultrasound images acquired **(A)** before ablation, **(B)** after HIFU initiated, **(C)** and **(D)** after further HIFU treatment, and **(E)** after ablation was completed. Images were acquired with LOGIQ E9 ultrasound machine and C1-6-D convex array probe from GE Healthcare after intravenous injection of 2.4 mL of SonoVue^®^ (sulphur hexafluoride microbubbles). Red arrows in the images indicate the volume that appeared devascularized after HIFU.
