# Supplementary figures and images for: Initial treatment for surgery-naïve desmoid tumors by high intensity focused ultrasound
Source: Front Oncol. 2024 Jul 22;14:1388302. doi: 10.3389/fonc.2024.1388302 (PMC11298426; doi:10.3389/fonc.2024.1388302)

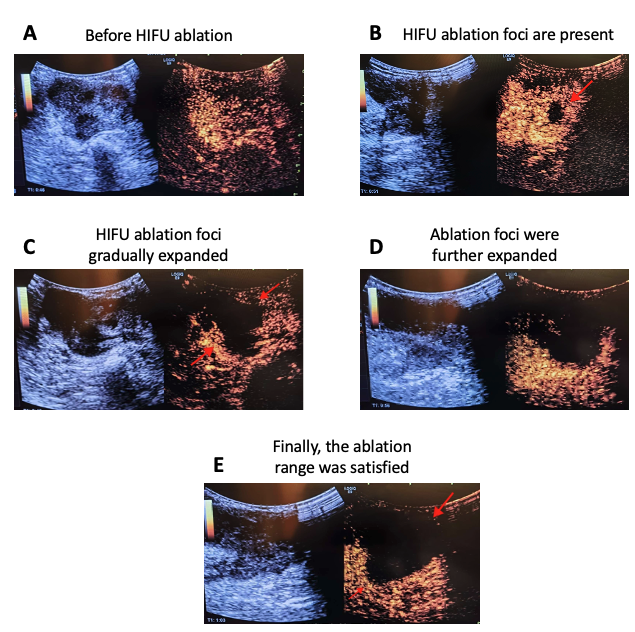

Supplement: Supplementary Figure 1 — Contrast-enhanced intraoperative ultrasound imaging used for intraoperative ablation monitoring. Ultrasound images acquired (A) before ablation, (B) after HIFU initiated, (C) and (D) after further HIFU treatment, and (E) after ablation was completed. Images were acquired with LOGIQ E9 ultrasound machine and C1-6-D convex array probe from GE Healthcare after intravenous injection of 2.4 mL of SonoVue® (sulphur hexafluoride microbubbles). Red arrows in the images indicate the volume that appeared devascularized after HIFU. [file Image_1.tiff]
